# A real-world, cross-sectional, and longitudinal study on high-risk human papillomavirus genotype distribution in 31,942 women in Dongguan, China

**DOI:** 10.3389/fpubh.2024.1409030

**Published:** 2024-08-01

**Authors:** Huanxia Zhong, Wenwei Pan, Binbin Chen, Jiamin Gu, Yu Liang, Guoli Sun, Xinghua Huang, Huitao Yuan, Haina Guo, Ling Zhong, Zhuanfen Li, Ping Zhou, Siliang Zeng, Li Tang

**Affiliations:** ^1^Women’s Health Department, Dongguan Maternal and Child Health Care Hospital, Dongguan, China; ^2^Department of Gynaecology, Dongguan Maternal and Child Health Care Hospital, Dongguan, China; ^3^BGI Genomics, Shenzhen, China; ^4^Health Department, Dongguan Maternal and Child Health Care Hospital, Dongguan, China; ^5^Cervical Clinic, Dongguan Maternal and Child Health Care Hospital, Dongguan, China; ^6^Pathology Department, Dongguan Maternal and Child Health Care Hospital, Dongguan, China; ^7^Medical Department, Dongguan Maternal and Child Health Care Hospital, Dongguan, China

**Keywords:** screening, high-risk human papillomavirus, cervical cytology, genotype distribution, infection rate, China, Dongguan

## Abstract

**Background:**

Persistent human papillomavirus (HPV) infection remains a key risk factor for cervical cancer. HPV-based primary screening is widely recommended in clinical guidelines, and further longitudinal studies are needed to optimize strategies for detecting high-grade cervical lesions compared to cytology.

**Methods:**

From November 2015 to December 2023, 31,942 participants were included in the real-world observational study. Among those, 4,219 participants underwent at least two rounds of HPV tests, and 397 completed three rounds of HPV tests. All participants were tested for high-risk types of HPV 16/18/31/33/35/39/45/51/52/56/58/59/66/68 (hrHPV) and low-risk types of HPV6/11 genotyping. Some participants also received cytology or colposcopy with pathology.

**Results:**

In the cross-sectional cohort, the prevalence of hrHPV and all HPV subtypes was 6.6% (2,108/31,942) and 6.8% (2,177/31,942), respectively. The three top hrHPV genotypes were HPV52 (1.9%), HPV58 (0.9%), and HPV16 (0.9%). Age distributions showed two peaks at 45–49 and 60–65 years. For the primary screening cohort, the hrHPV prevalence rate increased from 4.8% in 2015–2017 to 7.0% in 2020–2020 and finally reached 7.2% in 2023. For the longitudinal cohort study, the hrHPV prevalence rates in the repeated population (3.9, 5.3, and 6.0%) were lower than the primary hrHPV screening rates (6.6%), which indicated that repeated screening might decrease the prevalence rate. Methodologically, the hrHPV (89.5%) and the screening group of 16 subtypes (92.3%) demonstrated superior sensitivity than the cytology group (54.4%). Moreover, the longitudinal study indicated that the persistent hrHPV subgroup had a significantly higher (*p* = 0.04) incidence of high-grade squamous intraepithelial lesions and more histology progression events (7/17 vs. 0/5) than the reinfection group.

**Conclusion:**

The study indicates a rising high-risk HPV prevalence in Dongguan, with repeated screening reducing this trend. The findings support HPV-based primary screening and might guide HPV vaccination and cervical cancer prevention in South China.

## Introduction

According to the Global Cancer Observatory (GLOBOCAN) 2020 estimates, there were approximately 604,127 new cervical cancer cases and 341,831 deaths globally (1). The annual report of the China Cancer Registry indicated that both the incidence and mortality rates of cervical cancer increased in China in 2020, with an incidence rate of 11.35 and a mortality rate of 3.42 per 100,000 women (2). Further socioeconomic analysis indicates that as the human development index (HDI) increases, the incidence rates of cervical cancer tend to decrease (3). Additionally, various risk factors such as human papillomavirus (HPV) have also been associated with cervical cancer, especially the high-risk HPV (hrHPV) strains (4). There are more than 200 types of HPV, and approximately 14 hrHPV (HPV 16/18/31/33/35/39/45/51/52/56/58/59/66/68) strains are considered high risk for cervical cancer (4, 5).

The European Research Organization on Genital Infection and Neoplasia (EUROGIN) has recommended HPV testing as a primary screening approach for cervical cancer (6). The Addressing the Need for Advanced HPV Diagnostics (ATHENA) study, the largest prospective clinical study in the United States, assesses the use of HPV testing as the primary screening method for cervical cancer in women aged 25 years and older, which includes testing for hrHPV (14 pooled types) and genotyping for HPV 16 and HPV 18 (5, 7). Moreover, the American Society for Colposcopy and Cervical Pathology (ASCCP) guidelines also suggest that primary HPV testing screening should be considered as an alternative to the current cytology-based cervical cancer screening methods (8).

The HPV for cervical cancer (HPV FOCAL) trial reveals that multiple rounds of cytology missed more participants with cervical intraepithelial neoplasia grade 2 or higher (CIN2+) than HPV-based screening (9). The ATHENA study also confirms that primary HPV testing screening is safer and more effective than primary cytology-based screening (5, 7). In a real-world comparison conducted in Denmark, HPV-based screening demonstrated a 90% increase in the detection of cervical intraepithelial neoplasia grade 3 or higher (CIN3+) (10). Additionally, a cross-sectional study involving 11,064 Chinese women found that HPV-based screening with type 16/18 genotyping is more suitable for China due to a lack of cytologists in the country (11).

To address the high burden of cervical cancer in China, this longitude observational study aimed to evaluate the effectiveness of HPV testing with 14 high-risk genotyping and HPV6/11 types, alongside the ThinPrep cytologic test and colposcopy at the baseline or after repeat screening within 9 years. The goal was to provide evidence for the efficacy of repeated HPV-based screening in addressing the cervical cancer burden.

## Materials and methods

### Patients’ inclusion/exclusion criteria and relevant clinical data

A total of 31,942 women aged from 34 to 65 years old (y.o.) were finally enrolled in this observational real-world cervical cancer screening trial from November 2015 to December 2023. It is noteworthy that all of the data including the HPV test results and clinical information were collected in February 2024. Due to the policy support, all the clinical samples were collected by the clinician in the Dongguan Maternal and Child Health Care Hospital. Moreover, in this real-world single-center setting, the primary inclusion criteria included women aged between 34 and 65 years with a history of sexual activity. Those individuals with a history of hysterectomy or being pregnant or menstruating at the time of recruitment were excluded. Women of childbearing age must have adopted reliable contraceptive measures or have undergone a pregnancy test within 7 days before enrollment. The Dongguan Maternal and Child Health Care Hospital in Guangdong Province, China, was the candidate screening locale. Women who underwent the HPV test would receive the ThinPrep cytologic test and colposcopy examination based on individual preferences. Patients with cervical lesions were able to select the treatment options based on their actual clinical condition and personal preference. In our study, patients with cervical lesions were treated with physical therapy and excisional treatment. All collected clinical data at baseline and follow-up phases including screening date, patient age, HPV test results, cytology result, and pathology results are shown in Supplementary Table S1. Among those, the SeqHPV assay (BGI Shenzhen, PRC) on clinician-collected cervical samples was used for screening, and the detailed operating procedures were also shown in a previous publication (12).

### Study design

**Figure 1:**
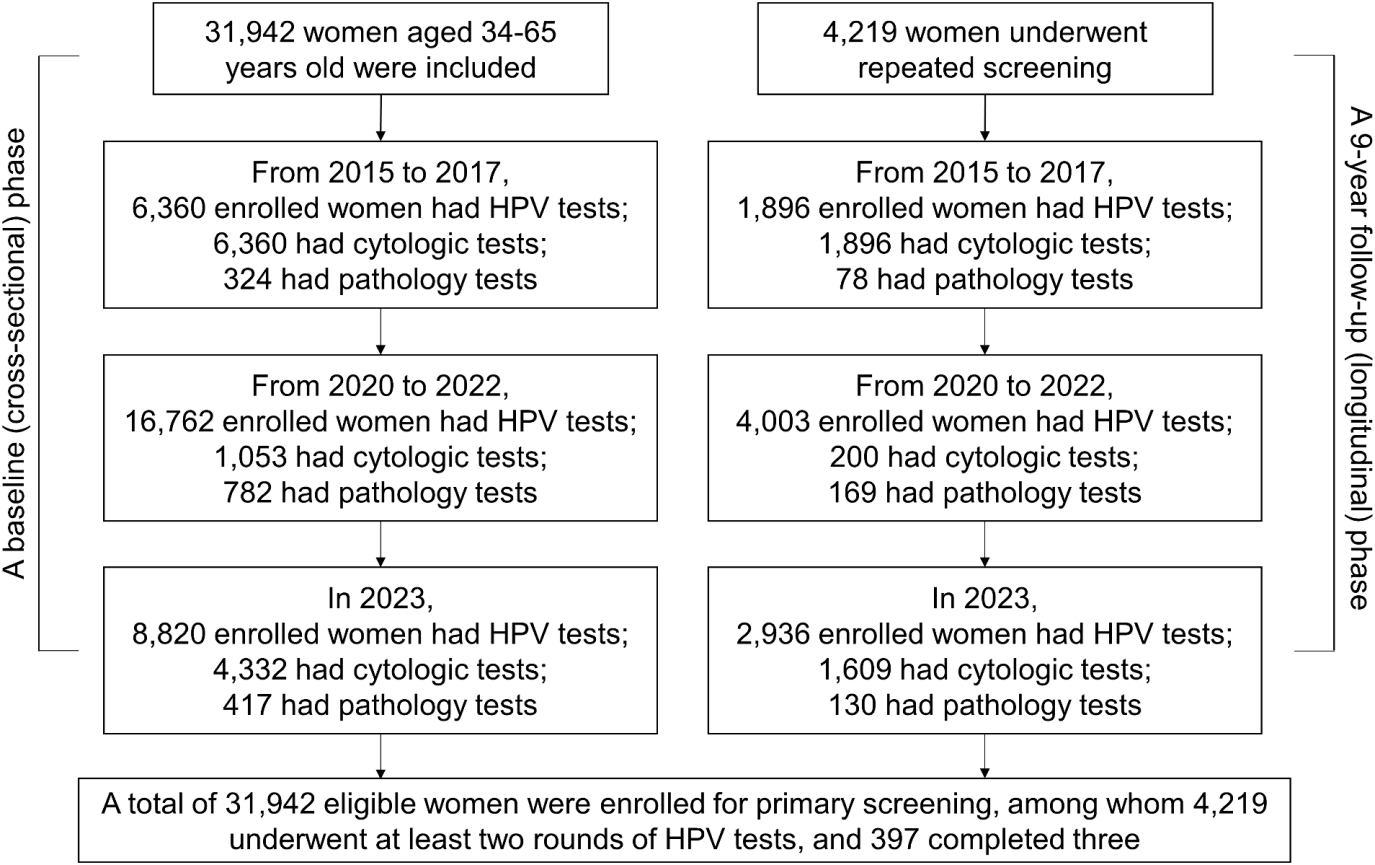
Timeline for the inclusion of the primary and repeated screening from 2015 to 2023.

The screening was conducted for three cycles from November 2015 to December 2023. However, the screening was temporarily stopped from 2018 to 2019 due to the shortage of medical resources and the outbreak of the Coronavirus disease 2019 (COVID-19) pandemic. The whole period was divided into three cycles: 2015–2017, 2020–2022, and 2023, with approximately 10,000 participants in each cycle. The timeline for the inclusion of the primary screening is shown in Figure 1. The study was conducted in two phases: a baseline (cross-sectional) phase and a 9-year follow-up (longitudinal) phase. For the baseline phase (cross-sectional phase), participating women were selected for primary screening as the baseline. For the follow-up phase (9-year longitudinal follow-up), women were scheduled for further second or third follow-up examinations from November 2015 to December 2023. It is noteworthy that all of the data including the HPV test results and clinical information were collected in February 2024.

### Statistical analysis

The prevalence of 14 hrHPV types (HPV 16/18/31/33/35/39/45/51/52/56/58/59/66/68) and all 16 HPV subtypes (14 hrHPV types plus low-risk types of HPV6/11) was indicated by positivity rates. The HPV infection and hrHPV infection in this study were defined as positive for all 16 HPV subtypes or 14 hrHPV subtypes, respectively. Abnormal rates of cytology and histology were calculated based on all participants with cytology and pathology testing results. The effectiveness of detecting abnormal histology was assessed by using the sensitivity and specificity between the three strategy groups (HPV, hrHPV, and cytology). All data were analyzed by SPSS 19.0 software or GraphPad Prism 9 software. A *p*-value of less than 0.05 (two-sided) was considered statistically significant.

## Results

### Clinical data and HPV prevalence among all eligible women

From November 2015 to December 2023, 31,942 women with a median age of 41 y.o. [aged 34–65 years, interquartile range (IQR): 37–46 years] were finally included in this trial. The detailed information of the clinical data is shown in Table 1. Most populations were within the 34–39 y.o. (41.9%) and 40–49 y.o. (41.8%) subgroups. For the 50–59 y.o. and 60–65 y.o. subgroups, the percentages were 14.1 and 2.2%, respectively. The overall positivity rate for the 16 HPV subtypes was 6.8%, with 2,177 out of 31,942 individuals testing positive. For hrHPV types, the positivity rate was slightly lower at 6.6% (2,108/31,942). In Table 2, the HPV prevalence results within different age groups presented two peaks, namely, a higher (45–49 y.o.) and a lower one (60–65 y.o.) both for the hrHPV and the HPV of 16 subtypes.

**Table 1:** Clinical data for all eligible women.

Characteristics	Eligible women (*n* = 31,942)
Age, y	
Mean ± SD	42.6 ± 6.7
34–39, *n* (%)	13,373 (41.9%)
40–49, *n* (%)	13,362 (41.8%)
50–59, *n* (%)	4,514 (14.1%)
60–65, *n* (%)	693 (2.2%)
HPV prevalence results	
HPV52	614 (1.9%)
HPV58	290 (0.9%)
HPV16	282 (0.9%)
hrHPV positive, *n* (%)	2,108 (6.6%)
HPV positive, *n* (%)	2,177 (6.8%)
Thinprep cytologic test	11,745
NILM	10,716
ASC-US	674
ASC-H	48
LSIL	219
HSIL	74
SCC	3
AGC	8
AGC, favor neoplastic	3
Pathological biopsy	1,523
Cervicitis	811
LSIL	496
HSIL	198
AIS	3
Carcinoma	15

AGC, atypical glandular cells; AIS, adenocarcinoma in situ; ASC-US, atypical squamous cells of uncertain significance; ASC-H, atypical squamous cells, cannot rule out HSIL; HPV: human papillomavirus; hrHPV; high-risk human papillomavirus; HSIL, high-grade squamous intraepithelial lesion; LSIL, low-grade squamous intraepithelial lesion; NILM, negative for intraepithelial lesions or malignancies; SCC, squamous cell carcinoma; SD, standard deviation.

**Table 2:** HPV prevalence was identified using the BGI HPV test.

		HPV positive, *n* (%)	
Age group, *y*	Total, *n*	HPV	hrHPV
34–39	13,373	878 (6.6%)	844 (6.3%)
40–44	8,441	593 (7.0%)	577 (6.8%)
45–49	4,921	353 (7.2%)	343 (7.0%)
50–54	2,836	172 (6.1%)	166 (5.9%)
55–59	1,678	122 (7.3%)	119 (7.1%)
60–65	693	59 (8.5%)	59 (8.5%)

HPV, human papillomavirus.

In Table 1, both the cytology and pathological tests were also performed for the included cohorts. In all, 11,745 individuals with ThinPrep cytologic test results and 1,523 participants with pathological biopsy results were recorded. From the cytological and pathological statuses, the recorded patients showed an abnormal cytology rate of 8.8% (1,029/11,745) and an abnormal histology rate of 46.7% (712/1,523).

### Human papillomavirus prevalence, cytology, and pathological status In three cycles of primary screening

The participants for primary HPV screening were 6,360, 16,762, and 8,820 within three cycles of 2015–2017, 2020–2022, and 2023, respectively (Figure 1; Table 3). A relatively small proportion with 29.8% (1,896 /6,360), 23.9% (4,003 /16,762), and 33.3% (2,936 /8,820) of individuals took repeated screening during the 1st, 2nd, and 3rd rounds, respectively. Moreover, in Table 3, both the HPV and hrHPV-positive rates gradually increased over time. For hrHPV types, the prevalence increased from 4.8% in 2015–2017 to 7.0% in 2020–2022 and finally increased to 7.2% in 2023. Similarly, the prevalence of 16 types of HPV increased from 5.1% in 2015–2017 to 7.1% in 2020–2022 and finally increased to 7.4% in 2023. In Table 4, the number of participants undergoing primary cytology screening was 6,360, 1,053, and 4,332 within three cycles of 2015–2017, 2020–2022, and 2023, respectively. The abnormal cytology rates were not gradually increased over time, with 5.5% in 2015–2017, 36.0% in 2020–2022, and 6.9% in 2023, which might be affected by the low population compared to HPV cohorts during COVID-19 (2020–2022). In Table 5, the number of patients with histology results was 324, 782, and 417 within three cycles of 2015–2017, 2020–2022, and 2023, respectively. For the pathology cohorts, the trends were similar to HPV cohorts, with the positive histological rates gradually increasing from 31.5% in 2015–2017 to 49.0% in 2020–2022 and finally increasing to 55.4% in 2023.

**Table 3:** HPV prevalence within the screening year.

		HPV positive, *n* (%)	
Screening year	Total, *n*	HPV	hrHPV
2015–2017	6,360	323 (5.1%)	308 (4.8%)
2020–2022	16,762	1,198 (7.1%)	1,166 (7.0%)
2023	8,820	656 (7.4%)	634 (7.2%)

HPV, human papillomavirus.

**Table 4:** Cytology results within the screening year.

		Thinprep cytologic test							
Screening year	Total, *n*	NILM	ASC-US	ASC-H	LSIL	HSIL	SCC	AGC	AGC, favor neoplastic
2015–2017	6,360	6,011	264	15	51	12	3	4	0
2020–2022	1,053	674	202	22	115	39	0	0	1
2023	4,332	4,031	208	11	53	23	0	4	2

AGC, atypical glandular cells; ASC-US, atypical squamous cells of uncertain significance; ASC-H, atypical squamous cells, cannot rule out HSIL; HSIL, high-grade squamous intraepithelial lesion; LSIL, low-grade squamous intraepithelial lesion; NILM, negative for intraepithelial lesions or malignancies; SCC, squamous cell carcinoma.

**Table 5:** Pathological results within the screening year.

			Pathological biopsy, *n* (%)			
Screening year	Total, *n*	Cervicitis	LSIL	HSIL	AIS	Carcinoma
2015–2017	324	222	55	37	0	10
2020–2022	782	399	275	102	3	3
2023	417	190	166	59	0	2

AIS, adenocarcinoma in situ; HSIL, high-grade squamous intraepithelial lesion; LSIL, low-grade squamous intraepithelial lesion.

### Human papillomavirus infection risk, cytology, and pathological results within three rounds of repeated screening

**Figure 2:**
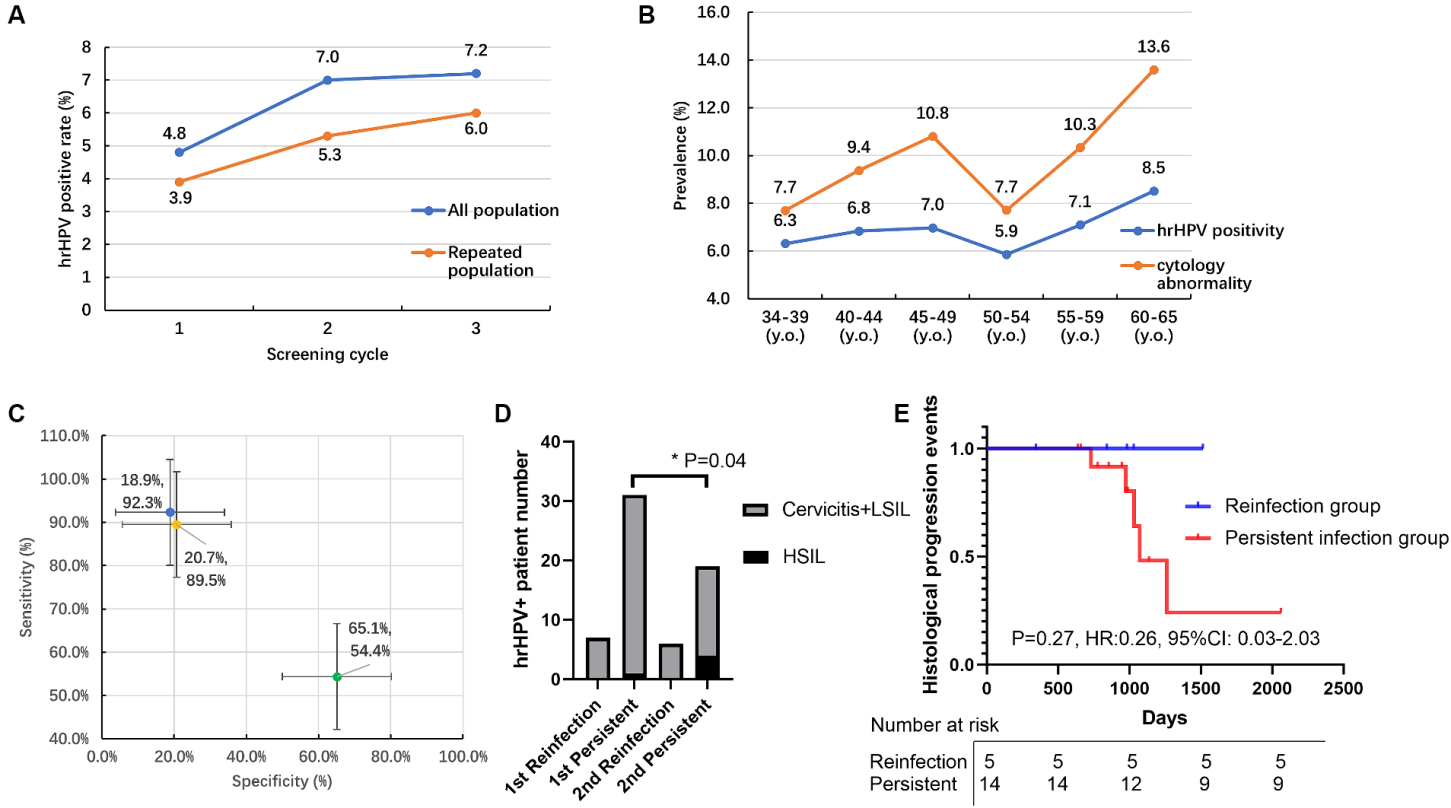
The HPV prevalence, cytology, and pathological results in the primary and repeated screening cohorts. **(A)** Prevalence of hrHPV positivity for all populations and repeated populations within three cycles of screening; **(B)** Prevalence of hrHPV positivity and cytology abnormality in different age groups; **(C)** Sensitivity and specificity of HPV (in blue), hrHPV (in yellow), cytology (in green) strategies to detect pathology abnormities; **(D)** The number of normal/abnormal histology patients for HSIL subtypes in persistent hrHPV infection versus reinfection groups within 1st and 2nd rounds of repeated screening. **(E)** The follow-up of increased histology events in persistent hrHPV infection and reinfection groups during screening. HSIL: high-grade squamous intraepithelial lesion. y.o., years old. *p < 0.05.

Except for the primary screening cohorts, 4,219 patients participated in at least two rounds of screening and were further analyzed as repeated screening subgroups (Table 6). In general, 4,219 individuals received two cycles and 397 individuals received three cycles of HPV screening. The prevalence of HPV in repeated subgroups demonstrated a trend similar to that observed in the primary screening cohort, with both HPV and hrHPV positivity rates exhibiting a gradual increase over time (Table 1; Figure 2A). For hrHPV, the positivity increased from 3.9% in the 1st screening to 5.3% in the 2nd screening and finally increased to 6.0% in the 3rd screening; for 16 types of HPV, the prevalence was also increased from 4.0 to 5.6% and finally increased to 6.2% for the 1st, 2nd, and 3rd rounds of screening, respectively. Interestingly, in Figure 2A, compared to primary screening cohorts, we observed that the hrHPV prevalence was slightly lower in repeated populations during the whole study. In addition, we also compared the hrHPV prevalence and cytologic abnormality in different age groups, as shown in Figure 2B. The results showed that the rate of cytology abnormalities was higher than hrHPV positivity in all age groups. Moreover, the two peaks for cytology abnormalities occurred at 60–65 y.o., with the lowest rate at 45–49 y.o., which was the same pattern observed with hrHPV. The study also compared the proportion of women referred for the ThinPrep cytologic test and colposcopy between HPV-based and cytology-based screening methods. We pooled the patients with histology results and compared the methodology of three screening strategies (HPV, hrHPV, and cytology), as shown in Figure 2C. The methodology analysis showed that the sensitivity for HPV (in blue), hrHPV (in yellow), and cytology (in green) strategies was 92.3, 89.5, and 54.4%, respectively. The specificity for HPV, hrHPV, and cytology cohorts was 18.9, 20.7, and 65.1%, respectively.

**Table 6:** HPV infection risk, cytology, and pathological results within three rounds of repeated screening.

		HPV positive, *n* (%)		Thinprepcytologic test								Pathological biopsy			
Screening round	Total, n	HPV	hrHPV	NILM	ASC-US	ASC-H	LSIL	HSIL	SCC	AGC	AGC, favor neoplastic	Cervicitis	LSIL	HSIL	Carcinoma
1st (15–17)	1,896	76 (4.0%)	73 (3.9%)	1,800	80	6	9	1	0	0	0	55	14	8	1
2nd (20–22)	4,003	223 (5.6%)	212 (5.3%)	127	42	2	24	4	0	0	1	90	64	15	0
3rd (23)	2,936	182 (6.2%)	175 (6.0%)	1,513	77	3	13	2	1	0	0	72	52	6	0

AGC, atypical glandular cells; ASC-US, atypical squamous cells of uncertain significance; ASC-H, atypical squamous cells, cannot rule out HSIL; HPV: human papillomavirus; hrHPV; high-risk human papillomavirus; HSIL, high-grade squamous intraepithelial lesion; LSIL, low-grade squamous intraepithelial lesion; NILM, negative for intraepithelial lesions or malignancies; SCC, squamous cell carcinoma.

Persistent type-specific HPV infection was defined as the detection of the same hrHPV type at baseline and the follow-up study (13). In our study, 60 individuals had positive hrHPV results within two cycles of screening, 83.3% (50/60) of the hrHPV-positive individuals presented with the same hrHPV subtype and were identified as persistent hrHPV infection within a screening interval of more than 3 years. The remaining 16.7% (10/60) of the reinfection group had a 28.6% (2/7) histology-positive rate in the 1st screening round, and this percentage decreased to 0% (0/6) in the 2nd screening round. For the persistent hrHPV infection subgroup, the histology-positive rate increased from 38.7% (12/31) to 52.6% (10/19), especially for the percentage of the high-grade squamous intraepithelial lesion (HSIL) percentage group, and this percentage significantly increased (*p* = 0.04) from 3.2% (1/31) to 21.1% (4/19), as shown in Figure 2D. According to the real-world hrHPV and histology data, the increased histology events only occurred (5/14) in the persistent infection group during a follow-up of more than 2000 days, and no events (0/5) occurred in the reinfection group (Figure 2E). More attention should be paid to the persistent hrHPV infection subgroup.

## Discussion

The prevalence of 14 hrHPVs and low-risk HPV types 6 and11 was analyzed in 31,942 Chinese women. Here, in our study, the prevalence of hrHPV was 6.6%, which was lower than the 12.6% hrHPV positivity rate reported in the ATHENA trial (5), as well as the 18.63% hrHPV prevalence documented in a separate study conducted in Guangzhou (14). This might be due to our limited inclusion criteria for participants of 34–65 y.o. in only one center. Moreover, we included individuals with a median age of 41 y.o. (IQR: 37–46), which was higher than these two studies. Actually, in our cohort, the hrHPV positivity of 6.6% was similar to another study with an overall HPV infection prevalence of 7.3% in the Guangdong province (15), which was lower than most other regions of China.

The prevalence of cytologic abnormalities was 8.8% in our study, which was higher than both the ATHENA study of 7.1% (5) and one multicenter randomized study of 5.9% with ASC-US+ (16). The elevated percentage observed may be influenced by the real-world data, which indicates that 93.2% (2029 out of 2,177) of HPV-positive patients underwent cytological examinations, in contrast to only 33.1% (9,860 out of 29,765) of HPV-negative individuals. Moreover, this phenomenon was more obvious in the subgroup with pathology results, in which 60.4% (1,315/2177) of HPV-positive and 0.7% (208/29765) of HPV-negative patients underwent pathology examination. The examination data were very low, especially during 2020–2022, which might be affected by both the COVID-19 pandemic and the shortage of medical resources. This data also reflected the real-world data that only the HPV-positive individuals would have further examinations. Moreover, due to the policy support (17), all participants underwent concurrent HPV and cytology tests during 2015–2017. There was a notable decline in the number of individuals receiving cytology tests during 2020–2022, which could be attributed to alterations in policy and the impact of the COVID-19 pandemic.

A previous study on Guangdong province has reported two peaks of HPV infection in the population of less than 25 y.o. (8.2%) and more than 50 y.o. (9.6%) (15). Another study in Guangzhou shows that two peaks were aged <21 and 46–50 years (14). A cross-sectional study presents two peaks: a higher (14–19 y.o.) and a lower one (30–34 y.o.) (18). In our cross-sectional cohort group, we only enrolled primary screening participants aged between 34 and 65 years, which might miss some crucial populations. Notably, we observed two age distribution peaks within the age ranges of 45–49 and 60–64 years, which was similar to previous reports of ages older than 46 years (14, 15). This pattern might be attributed to geographical variations, as our cohort was exclusively drawn from patients at a single hospital in Dongguan. It was also important to highlight that the representation at the extreme ends of our age spectrum was minimal, with only 1 patient aged 34 years and 3 patients aged 65 years. Such low numbers at these points were insufficient for statistical analysis to draw reliable conclusions. Moreover, the peaks for cytologic abnormalities were at 45–49 and 60–64 y.o., which was the same as the phenomenon in the hrHPV group, confirming the real-world data of both hrHPV and the cytology procedure.

Our longitude cohort study had a follow-up of approximately 9 years for a repeated population of 4,219 participants. The hrHPV prevalence was also increased in this repeated screening subgroup over time. While compared to the primary screening cohort, the total percentage was lower than the average percentage of 6.6% hrHPV prevalence rate. One study shows that the negative conversion rate of hrHPV increased to 68.9% within half a year and increased most rapidly within the first 2 years after treatment (19). Another study shows that all subtypes of HPV-negative rates were 81.81, 85.71, and 90.91% at 6, 12, and 24 months, respectively (20). Factors such as age, HPV type, sexual behavior, and initial treatment can affect HPV clearance, yet most HPV-infected women typically remove the virus within 6 to 12 months (21). In our cohort, our hrHPV-negative rate was only 45.8% (50/76) between primary screening and the second screening rounds, which might be affected by our long follow-up of more than 3 years. Additionally, the histology-positive rate significantly increased from 38.7 to 52.6% in the persistent hrHPV infection group, especially for the percentage of HSIL, which significantly increased from 3.2 to 21.1%. The recent longitudinal study, including a multicenter cohort study, has demonstrated the reliable efficacy of a real-time polymerase chain reaction (PCR)-based assay in cervical screening (22). This assay differentiates between HPV16 and HPV18 and 12 other hrHPV types among 9,829 eligible women, with a follow-up of 3 years (22). Another 3-year longitudinal study of 10,186 women reveals that infections with hrHPV types other than HPV 16/18 are responsible for a large number of CIN2 and CIN3+ cases (23). In our study, we conducted a 9-year follow-up involving larger populations. Additionally, by utilizing next-generation sequencing (NGS) techniques, we achieved a high-throughput analysis of 16 specific HPV subtypes.

As this is a retrospective real-world study, certain limitations are inherent, including the use of specific inclusion and exclusion criteria. Patients could choose the treatment options based on their actual clinical condition and willingness. This approach slightly differs from that of the ATHENA study, which indicated that patients did not have prearranged plans for further HPV treatment (5). In our real-world cohort, patients with cervical lesions had physical therapy or excisional treatment. Figure 1 shows the number of participants screened during each cycle, with re-screening based on individual willingness. Consequently, a relatively small proportion of individuals of 29.8, 23.9, and 33.3% underwent repeated screening during the 1st, 2nd, and 3rd rounds, respectively. The slight decrease in participation during the second round from 2020 to 2022 might have been influenced by the COVID-19 pandemic. There is no definitive treatment for HPV infection to date; emerging immunotherapeutic approaches, such as toll-like receptor agonists, therapeutic HPV vaccines, and immune checkpoint inhibitors are under investigation (24). The routine treatment could not guarantee HPV clearance. Other studies on the gene polymorphisms of toll-like receptors and cyclooxygenase-2 in relation to cervical cancer risk hold promise for advancing our understanding of predictive biomarkers, which could significantly enhance the risk stratification for hrHPV (25, 26). Further studies could explore future predictive biomarker development, as well as the pathological outcomes and treatment responses associated with these HPV subtypes, monitored over the course of the follow-up period.

A recent study focusing on primary cervical cancer screening in Latin America has shown that the sensitivity of CIN3+ using cytology and HPV tests was 48.5 and 98.1%, respectively (27). The results were similar to our subgroup with histology results; we concluded that the sensitivity was very high in both the hrHPV screening group (89.5%) and the 16 subtypes group (92.3%) compared to the cytology group (54.4%). The specificities observed in our three groups were notably lower than the reported 96.5% for cytology and 88.7% for HPV testing (27), with our figures being 20.7% for the hrHPV group, 18.9% for the 16 HPV subtypes group, and 65.1% for the cytology group. This discrepancy may be due to the high proportion of HPV-positive individuals in our study cohort, where 93.2 and 60.4% of HPV-positive patients underwent cytological and pathological examinations, respectively, compared to only 33.1 and 0.7% of HPV-negative individuals. In our study, the rapid and low-cost isothermal amplification HPV assay (12), which is priced at half the cost of the ThinPrep cytologic test, could also be recommended because of its high sensitivity and high-throughput characteristics. The findings from this study have the potential to guide future HPV immunization strategies and the development of policies aimed at preventing cervical cancer in Dongguan.

## Conclusion

In this real-world study for hrHPV screening among 31,942 Chinese women, the prevalence of hrHPV was 6.6% in the cross-sectional cohort. We also observed two age distribution peaks within the age ranges of 45–49 and 60–64 years. For the longitude cohort study, a repeated population of 4,219 participants with a follow-up of nearly 9 years was investigated. The hrHPV prevalence was decreased in the repeated screening cohort than in the primary screening cohort. It is noteworthy that within the cohort subjected to repeated screening, the subgroup with persistent hrHPV infections exhibited a higher HSIL-positive rate and more histological progression events than the hrHPV reinfection subgroups. Furthermore, the sensitivity of the hrHPV screening group (89.5%) and the 16 subtypes group (92.3%) surpassed that of the cytology group (54.4%). Despite the HPV test having a lower specificity and higher cost compared to cytology, the HPV test should also be considered due to its superior sensitivity and high-throughput capability.

## Data availability statement

The original contributions presented in the study are included in the article/Supplementary material, further inquiries can be directed to the corresponding author/s.

## Ethics statement

This study was approved by the Ethics Committee of Dongguan Maternal and Child Health Care Hospital (Ethics approval number: Dongguan Maternal and Child Health Care Hospital-2022-103) and the BGI Genomics (grant no. BGI-IRB 24029). The studies were conducted in accordance with the local legislation and institutional requirements. The participants provided their written informed consent to participate in this study.

## Author contributions

HZ: Data curation, Formal analysis, Funding acquisition, Writing – original draft, Writing – review & editing. WP: Data curation, Formal analysis, Writing – original draft, Writing – review & editing. BC: Data curation, Formal analysis, Writing – original draft, Writing – review & editing. JG: Data curation, Validation, Writing – original draft, Writing – review & editing. YL: Data curation, Writing – original draft, Writing – review & editing. GS: Data curation, Writing – original draft, Writing – review & editing. XH: Data curation, Writing – original draft, Writing – review & editing. HY: Data curation, Writing – original draft, Writing – review & editing. HG: Data curation, Writing – original draft, Writing – review & editing. LZ: Data curation, Writing – original draft, Writing – review & editing. ZL: Data curation, Writing – original draft, Writing – review & editing. PZ: Data curation, Supervision, Writing – original draft, Writing – review & editing. SZ: Data curation, Supervision, Writing – original draft, Writing – review & editing. LT: Data curation, Supervision, Writing – original draft, Writing – review & editing.
